# Potentiation of IL-4 Signaling by Retinoic Acid in Intestinal Epithelial Cells and Macrophages—Mechanisms and Targets

**DOI:** 10.3389/fimmu.2020.00605

**Published:** 2020-05-05

**Authors:** Celine Chen, Allen D. Smith, Lumei Cheung, Quynhchi Pham, Joseph F. Urban, Harry D. Dawson

**Affiliations:** Diet, Genomics, and Immunology Laboratory, Beltsville Human Nutrition Research Center, Agricultural Research Service, USDA, Beltsville, MD, United States

**Keywords:** macrophage, porcine, human, interleukin 4, all-trans-retinoic acid

## Abstract

We previously demonstrated that IL4, IL13, CLCA1, and CCL26 mRNA were significantly upregulated in the lungs of pigs given a low dose of all trans-retinoic acid (ATRA) and infected with Ascaris suum. We also demonstrated that *in vitro* ATRA induced a state of partial alternative activation in porcine macrophages (Mφs) and amplified certain aspects of M2a activation induced by IL-4. Given these results, we tested the effect of ATRA on IL-4 responses in two porcine intestinal epithelial cell lines, IPEC1 and IPEC-J2 and observed that ATRA increased mRNA for the IL-4 receptor alpha chain. ATRA also increased IL-4 induced phosphorylation of signal transducer and activator of transcription 6 (STAT6) and mRNA expression of the chloride channel, calcium activated, family member 1 (CLCA1), important for mucus formation, and chemokine (C-C motif) ligand 26 (CCL26), a potent eosinophil chemoattractant. We extended these findings to human Mφ THP-1 cells and showed that ATRA synergistically increased IL-4–induced CCL2, CCL13, and CCL26 mRNA and protein levels. Transglutaminase 2 mRNA, protein, and enzyme activity were synergistically induced in THP-1 cells pretreated with ATRA and then treated with IL-4, thus, ATRA increased signaling in response to IL-4 in porcine epithelial cells and porcine and human Mφs. Given the prevalence of allergic and parasitic diseases worldwide and the close similarities in the porcine and human immune responses, these findings have important implications for the nutritional regulation of allergic inflammation at mucosal surfaces.

## Introduction

Vitamin A (VA) is required for T helper 2-associated responses that are shared by immune responses to allergens and parasites (1–3). In mice, VA deficiency reduced (3) or increased (4) pulmonary Th2 immune responses to experimental pulmonary allergy models. Similarly, supplemental VA or ATRA increased (3, 5) or decreased (6) pulmonary Th2 responses. These effects seem to be timing and dose dependent. *In vivo* allergic responses in pigs are increased by ATRA in response to parasitic infections including increased lung eosinophilia and expression of Th2-associated cytokines, interleukin 4 (IL-4) and IL13, eosinophil chemo-attractants (Chemokine (C-C motif) ligand 11 (CCL11), CCL22, CCL17, and CCL26) and the goblet cell differentiation marker, chloride channel, calcium activated, family member 1 (CLCA1) (7).

Macrophages are one likely target of VA in these models. Polarized Mφs are important for regulating distinct immune responses in tissues. The most actively studied are classically activated (M1) which mediate host defense leading to type I inflammation or alternatively activated M2 which have been divided into several subtypes. Alternatively activated Mφs (AAM) stimulated by interleukin 4 (IL-4) and IL-13 are designated as M2a that can suppress inflammation and promote wound healing and are dependent upon the transcription factors signal transducer and activator of transcription 6 (STAT6) and interferon regulatory factor 4 (IRF4) for their development (8–11). Type-II AAMs, known as M2b, mediate immune complex immunoregulation and Th2 activation, and M2c are stimulated by IL-10 and glucocorticoids to primarily modulate tissue remodeling (8, 10, 11).

M1, M2a, or M2b differentiation can be influenced by dietary factors. ATRA, the active component of VA, generally inhibits the development of M1 macrophage. Thus, ATRA inhibited LPS-induced TNF, CCL3, and CCL4 mRNA and protein levels in human primary Mφs and THP-1 cells (12, 13); but had no effect on IL1A or IL1B mRNA (13). The mechanisms behind these phenomena are partially known. ATRA stimulated LPS-induced production of the anti-inflammatory cytokine, IL-10 (12, 13). In addition, ATRA-inhibited basal and LPS- or TNF-induced NF-κB activation (14–16). Whether these mechanisms extend to ATRA-induced differentiation of human M2a cells is unknown.

Several recent reports suggested that ATRA can influence M2a or M2b Mφ development. Lee et al. demonstrated that ATRA increased IL-4 induced arginase 1 (Arg1) mRNA, protein expression, and enzymatic activity in mouse RAW264.7 Mφs (17). However, it had no effect on IL-4 induced mannose receptor; C type 1 (Mrc1) or chitinase 3-like 3 (Chi3l3/Ym1) mRNA expression. Two separate groups demonstrated that ATRA increased Arg1 protein expression in mouse bone marrow-derived Mφs (18, 19), and Gundra et al. demonstrated that VA was necessary for M2a development in *Schistosoma* infected mice (20).

Transglutaminase 2 (TGM2), one of the few, *trans*-species markers for M2a Mφs (21), can be induced in human Mφs treated with ATRA (22). We recently demonstrated that ATRA- induced mRNA for CCL17 and CCL22 in pig primary alveolar Mφs and a pig alveolar Mφ cell line, and synergized with IL-4 to induce mRNA for all of the major eosinophil chemoattractants, CCL11, CCL17, CCL22, and CCL26 (7). Similarly, ATRA synergistically enhanced IL-4 up-regulation of TGM2 at the mRNA and protein level (23). There are a limited number of studies showing the effect of ATRA on human M2b Mφ polarization. Treatment of human-derived THP-1 cells with the combination of ATRA and 1,25 (OH)_2_ vitamin D3 (VD3) increased CD163 (an M2b marker), ARG1 and IL10 TGFB1 mRNA and increased clusters of differentiation 163 (CD163) and IL-10 protein expression (24).

Epithelial cells also participate in the immune response to helminths. Intestinal epithelial tuft cells by virtue of their secretion of IL-25, IL-33 and thymic stromal lymphopoietin (TSLP) initiate type 2 mucosal immunity (25). These cytokines result in IL-13 production by type 2 innate lymphoid cells (ILC2). Nutritional or metabolic factors that influence this axis include succinate (26), vitamin D (27) and vitamin A, via its conversion to ATRA (27, 28). Il-4 and IL-13 have pleitropic effects on epithelial cells including induction of CLCA1 (29), eosinophil-attracting chemokines (CCL17 (30), CCL24 (31), and CCL26 (31). Vitamin A is essential for normal epithelial cell differentiation and function including goblet cell development, fluid and mucus production (32, 33). The effects of ATRA on epithelial cell responses to IL-4/IL-13 have not been extensively studied. ATRA decreased CCL24 and increased CCL26 protein production from IL-4 stimulated human primary bronchial epithelial cells (31).

Nutritionally-based pig models have served as an important translational bridge between research findings made in rodents and humans. Recent comparative structural and functional genomics-based analysis of the mouse, human and pig genomes (34–37) reinforced this concept and extended it to inflammatory and immune responses. One of our goals in the current series of studies is to define the role of ATRA in mediating epithelial cell responses in response to IL-4 and IL-13 in 2 non-transformed porcine epithelial cell lines.

Herein, we demonstrate that ATRA induces IL-4 receptor mRNA and that ATRA-treatment of IPEC-1 and IPEC-J2 porcine epithelial cells led to greater IL-4-induced STAT6 phosphorylation and CLCA1 mRNA was induced in a dose-dependent fashion by ATRA, IL-4, or IL-13. Our data also indicates that, like porcine Mφs, porcine intestinal epithelial cell responses to IL-4 are increased by exposure to ATRA. ATRA synergistically increased IL-4–induced CCL2, CCL13, and CCL26 mRNA and protein levels in human MφTHP-1 cells. Transglutaminase 2 mRNA, protein and enzyme activity were synergistically induced in THP-1 cells pretreated with ATRA and then treated with IL-4. Thus, in porcine epithelial cells and porcine and human Mφs, ATRA increased signaling in response to IL-4 suggesting that ATRA treatment induces a conserved response across cell types and species.

## Materials and Methods

### Cell Culture

IPEC-1 cells were provided by Dr. Dennis Black, Department of Pediatrics and the Department of Medicine, University of Tennessee Health Science Center, Memphis, Tennessee. IPEC-J2 cells were provided by Dr. Sean Bearson, NADC, Ames Iowa. IPEC-1 and IPEC-J2 cells were incubated at 37°C in an atmosphere containing 5% CO2 in humidified air. Undifferentiated IPEC-1 or IPEC-J2 cells were maintained in serial passage in plastic culture flasks (75 cm^2^, Corning, Corning, NY) in Dulbecco's Modified Eagle's Medium (DMEM)/F12 medium (Gibco Invitrogen Corporation, Grand Island, NY) supplemented with 5% fetal bovine serum (FBS) (Hyclone South Logan, UT), 5 μg/ml ITS Premix® (insulin 5 ug/ml, transferrin 5 ug/ml, selenium 5 ng/ml, BD Biosciences, Bedford, MA), 5 μg/ml epidermal growth factor (BD Biosciences), penicillin (50 μg/ml), and streptomycin (4 μg/ml) (QBI, Gaithersburg, MD) (growth medium). Adherent undifferentiated cells were washed 2 times with PBS without Ca^2+^ or Mg^2+^ and harvested by treatment with 5 mls of TrypLE™ Select (Gibco Invitrogen) enzymatic dissociation media and gentle scraping. Cells were washed 2 times with PBS without Ca^2+^ or Mg^2+^ and re-suspended at 1 X 10^6^ cells/ml in growth media. Two mls of this suspension was plated in each well of a 6 well plate (Corning) for 12 h. Depending on the experiment, IPEC-1 cells were treated with 0.5 to 50 ng/ml of recombinant porcine IL-4 (Invitrogen, Camarillo, CA) or human IL-13 (Invitrogen) for 24 or 48 h to determine the half maximal effective concentration (EC50). For experiments involving ATRA, cells were treated with EtOH, or 0.1 to 1,000 nM ATRA (Sigma) in EtOH. Cells were pre-treated with 10^−7^ M ATRA 24 h before exposure to 5 ng/ml of IL-4 for another 24 h where indicated.

THP-1 cells were obtained from ATCC (Manassas, VA) and cultured in RPMI (Quality Biological, Gaithersburg, MD) supplemented with 10% heat-inactivated fetal bovine serum (Gibco, Gaithersburg, MD), 1% penicillin/streptomycin (Quality Biological, Gaithersburg, MD), and 1% sodium bicarbonate (Quality Biological, Gaithersburg, MD). Prior to treatments, cells were seeded at 5 × 10^5^ cells/ml and differentiated with 25 ng/ml phorbol-12-myristate-13-acetate (PMA, EMD-Calbiochem, La Jolla, CA) for 48 h. After differentiation, cells were treated with EtOH or ATRA (100 nM), Sigma, St. Louis, MO) or recombinant human IL-4 (10 ng/ml, Leinco Technologies, St. Louis, MO) or ATRA + IL4 or LPS from *Escherichia coli* 0111: B4 strain (10 ng /ml, InvivoGen, San Diego, CA). Supernatants and cellular RNA and protein were harvested at 1, 2, 4, 8, 24, or 48 h. In some experiments VD3 (Sigma) in EtOH was added at a final concentration of (10 nM), with or without (100 nM) ATRA.

### NFκB-Reporter Assay

The THP1-XBlue™ cell line and QUANTI-Blue reagent were obtained from InvivoGen (San Diego, CA). The NF-κB/AP-1 reporter assay was carried out following the manufacture's protocol. The THP1-X Blue cells were seeded at 1 × 10^6^ cells/ml (180 μL per well) in a 96 well plate and cultured at 37°C in 5% CO_2_. Cells were differentiated and then pretreated with EtOH or ATRA, as described above. Cells were then treated with IL4 or LPS for 24 h as described above. Mock-transfected THP-1 cells were used as controls. The supernatants from these cell culture samples were then mixed with the QUANTI-Blue solution (as described by the manufacture's product data sheet) for 4 h. Secreted embryonic alkaline phosphatase (SEAP) levels were determined by a spectrophotometer at 620–655 nm.

### Real-Time PCR

For IPEC cells, supernatants were discarded, and cells were washed two times with 1X PBS without Ca2+ or Mg2+. Adherent cells were then homogenized in 2 ml of TRIzol (Invitrogen, Carlsbad, CA) for RNA extraction or processed for analysis by flow cytometry. RNA extraction, cDNA synthesis and real-time PCR analysis was essentially as described (8). For each message, the fluorescence signals measured during amplification were processed post-amplification and the value (Ct) calculated when the fluorescence intensity was 20-fold greater than the standard deviation of the baseline fluorescence. All mRNA levels were normalized to PPIA by subtracting its Ct value from the Ct value for each message. This value was defined as the adjusted (Adj) Ct. The means of the Adj Ct were compared to the means of the control and fold changes were calculated to be 2^AdjCt^. For THP-1 cells, all mRNA levels were normalized to PPIA and compared to untreated or EtOH-treated controls where indicated. RNA extraction, cDNA synthesis and real-time PCR analysis was essentially as described [(23) #4380]. All gene sequence information including primer and probe sequences can be found in the Porcine Translational Research Database (http://www.ars.usda.gov/Services/docs.htm?docid=6065).

### ELISA Analysis of Cytokines

Human CCL2 (R & D Systems # DCP00), CCL13 (Thermo Scientific #), CCL17 (Thermo Scientific # EHCCL17), CCL18 (R & D Systems), CCL21 (R & D Systems # DY366), CCL22 (Thermo Scientific #), CCL26 (Raybiotech # ELH-Eotaxin3), IL1B (Invitrogen # KHC00012), IL1RN (R & D Systems # DRA00B), and TNF (R & D Systems # DTA00D) were measured in cell culture supernatants by ELISA, according to the manufacturer's instructions. Results were obtained using a Spectramax 96 well Spectrophotometer.

### Western Blot Analysis of IRF4 and IL-4 Receptor Proteins

Cells were obtained and cultured in 6-well plates as described above. Cells were treated with ATRA (100 nM) for 24 h and then IL-4 (5 ng /mL). After 24 h, wells were washed 2 X with PBS (w/o Ca^2+^ and Mg^2+^) and treated with 0.8 mL of MPER reagent (Thermo-Fisher Scientific). Protein measurements on lysates were made using the Pierce BCA Protein Assay Kit (Thermo-Fisher Scientific).

For IRF4 determination, samples (10 μg) or control IRF4 (LSBio # G76848-20) lysate were subjected to reducing Tris-Glycine SDS-gel electrophoresis on 10–20% polyacrylamide gels (Novex, Invitrogen, Carlsbad, CA). The gels were electroblotted onto nitrocellulose using a Tris/Glycine transfer buffer (Novex) with 20% methanol added. For IL-4 receptor detection, gels were run under non-reducing conditions using IL-4R transfected cells control lysates (LSBio # G76916-20). The resulting blots were blocked with Odyssey Blocking buffer (LI-Cor Biosciences, Lincoln, NE) at room temperature for 1 h and then incubated with anti-human IRF4 polyclonal antibody [Santa Cruz (SC-48338), 1:200)] anti-human IL-4R monoclonal antibody 25463 (R & D Systems # MAB230100 2 1:500) and rabbit anti-GAPDH (Cell Signaling # 21185, 1:1000-1:2000) overnight at 4°C. Blots were then washed with PBS/0.1% Tween-20 and incubated with Li-Cor IRDye 800CW labeled Goat anti-rabbit IgG (Catalog # 926-32211) for 2 h at room temperature, washed with PBS/0.1% Tween-20 and imaged using the Odyssey CLx Imaging System (LI-Cor Biosciences, Lincoln, NE). Bands were quantitated using the Li-Cor Image Studio Software. The ratio of IRF4 or IL-4R signal to GAPDH signal was calculated.

### Transglutaminase 2 ELISA and Enzymatic Assay

Protein lysates were prepared as described above and 20 μL of lysate was used per well. TGM2 protein was quantitated by an ELISA kit (Sigma # RAB1741). Transglutaminase enzymatic activity was quantitated by a colorimetric assay (Novus Biologicals # NBP1-37008). Absorbance was measured using a Spectramax Microplate Reader (Molecular Devices, LLC, San Jose, CA) using a wavelength of 450 nm. Transglutaminase activity was expressed as mU/ug protein.

### Statistics

All statistical analysis was performed using JMP (v12.0 SAS Institute Inc., Cary NC). Data were analyzed for equality of variance using Fisher's F test. Messenger RNA expression (ΔCt values), protein levels and STAT6 fluorescence values were evaluated by a Student *t*-test and/or a one-way ANOVA where indicated. The Fisher's Least Squares Difference *post-hoc* test was applied to assess differences between treatment groups. For all analyses, *p*-values < 0.05 were considered significant.

## Results

Previously we observed that IL4, IL13, CLCA1, and CCL26 mRNA were significantly up regulated in the lungs of *A. suum*-infected pigs given low dose of ATRA (7), indicating that ATRA enhanced the parasite-induced Th2 response. However, lung tissue is composed of multiple cell types including Mφs and epithelial cells. We wished to know whether lung epithelial cells contributed to the enhanced accumulation of eosinophils in the lungs of the animals infected with Ascaris and given ATRA. As there are no porcine lung epithelial cell lines available and Ascaris also induced a Th2 response in the ileum of infected animals (38), we examined the effect of ATRA on IL-4 induced gene expression in two porcine, non-transformed, small intestinal epithelial cell lines, IPEC-1 and IPEC-J2. Because of potential expression differences between cell lines, we first determined whether a panel of genes including, CLCA1 or CCL26 were IL-4 and IL-13 responsive in porcine epithelial cells. Our results (Table 1) showed that both genes were induced in IPEC-1 cells by IL-4 and IL-13 in a dose dependent manner with a half maximal effective concentration (EC50) estimated to be 5–10 ng/mL.

**Table 1:** IL-4 and IL-13 increases IPEC-1 cell CLCA1 and CCL26 mRNA expression in a dose-dependent fashion.

		**Mean Adj. CT ± SD**	**Fold change vs. control**	**Significance**
**(A) IL-4**				
CLCA1	Control	16.5 ± 0.0	–	–
	0.5 ng/ml	16.9 ± 0.2	1.3	–
	1.0 ng/ml	17.2 ± 0.3	1.6	0.07
	5.0 ng/ml	18.1 ± 0.2	3	0.007
	10 ng/ml	18.4 ± 0.6	3.7	0.002
	50 ng/ml	18.3 ± 0.5	3.5	0.002
CCL26	Control	0.0 ± 0.0	–	–
	0.5 ng/ml	0.4 ± 0.5	1.3	–
	1.0 ng/ml	1.4 ± 0.7	2.6	0.02
	5.0 ng/ml	3.5 ± 0.9	11.3	<0.0001
	10 ng/ml	4.5 ± 0.4	22.4	<0.0001
	50 ng/ml	4.2 ± 1.1	17.1	<0.0001
**(B) IL-13**				
CLCA1	Control	16.5 ± 0.2	–	–
	0.5 ng/ml	17.9 ± 0.6	2.6	0.002
	1.0 ng/ml	18.1 ± 0.5	3.2	0.0007
	5.0 ng/ml	18.8 ± 0.4	4.9	<0.0001
	10 ng/ml	20.0 ± 0.6	11.3	<0.0001
	50 ng/ml	20.5 ± 0.1	17.1	<0.0001
CCL26	Control	0.8 ± 0.6	–	–
	0.5 ng/ml	2.9 ± 0.9	4.3	0.008
	1.0 ng/ml	3.6 ± 0.9	7.0	0.001
	5.0 ng/ml	4.0 ± 0.6	9.2	0.0005
	10 ng/ml	5.3 ± 1.1	22.6	<0.0001
	50 ng/ml	6.7 ± 1.0	59.7	<0.0001

*The swine intestinal epithelial cell line IPEC-1 was treated with the indicated doses of porcine IL-4 or human IL-13 for 24 h. Messenger RNA of CLCA1 was analyzed as indicated in materials and Methods using RPL32 as an internal housekeeping gene. Data were compared by ANOVA. [2 technical replicates, (n = 4 per group)]*.

We next wanted to determine whether these genes responded to ATRA in a dose-and time (1, 2, 4, 8, 24, 48, and 72 h) dependent fashion. Cytochrome P450, family 26, subfamily A, polypeptide 1 (CYP26A), a retinoic acid-inducible gene, was used as a control. Only CYP26A and CLCA1 mRNA were induced by ATRA in a dose and time-dependent fashion (data not shown) with a maximum effective concentration of ATRA of 10^−7^ M. The optimal incubation time period was 24 h.

**Figure 1:**
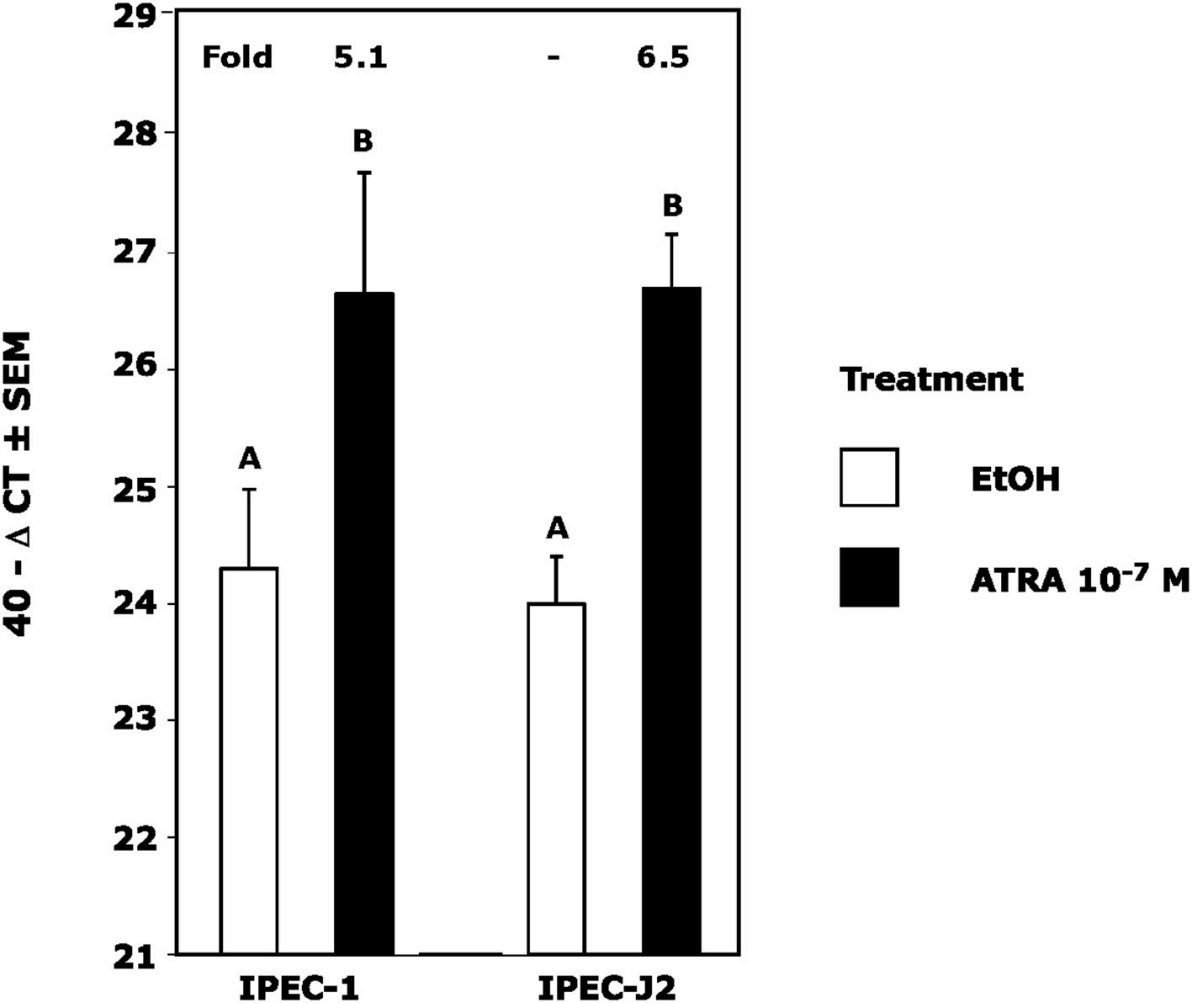
ATRA Upregulates IL4 Receptor RNA in IPEC1 and IPEC-J2 Cells. IPEC1 or IPEC-J2 cell lines were treated with ± EtOH or 100 nM ATRA for 18 h. CLCA1 mRNA was analyzed as indicated in Materials and Methods using RPL32 as an internal housekeeping gene. Data were compared by student *t*-test. Means with non-matching superscripts are significantly different at *p* < 0.005 [(*n* = 4 per group), 1 technical replicate].

**Figure 2:**
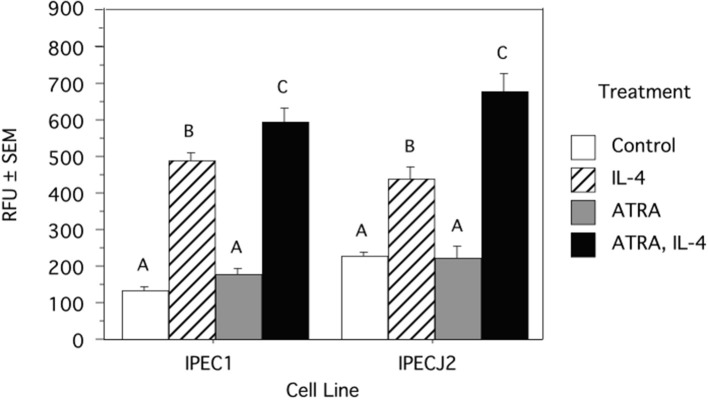
ATRA Increases IL-4 Induced STAT6 Phosphorylation in IPEC1 and IPEC-J2 Cells. IPEC1 or IPEC-J2 cell lines were treated with ± EtOH or 100 nM ATRA for 18 h and then treated with 5.0 ng/ml of porcine IL-4 for 10 min. Phosphorylation was determined by a cell-based ELISA (R and D Systems). Data were compared by ANOVA and are expressed as arbitrary fluorescent units. Means with non-matching superscripts are significantly different at *p* < 0.05 [(*n* = 3 per group), 1 technical replicate].

**Figure 3:**
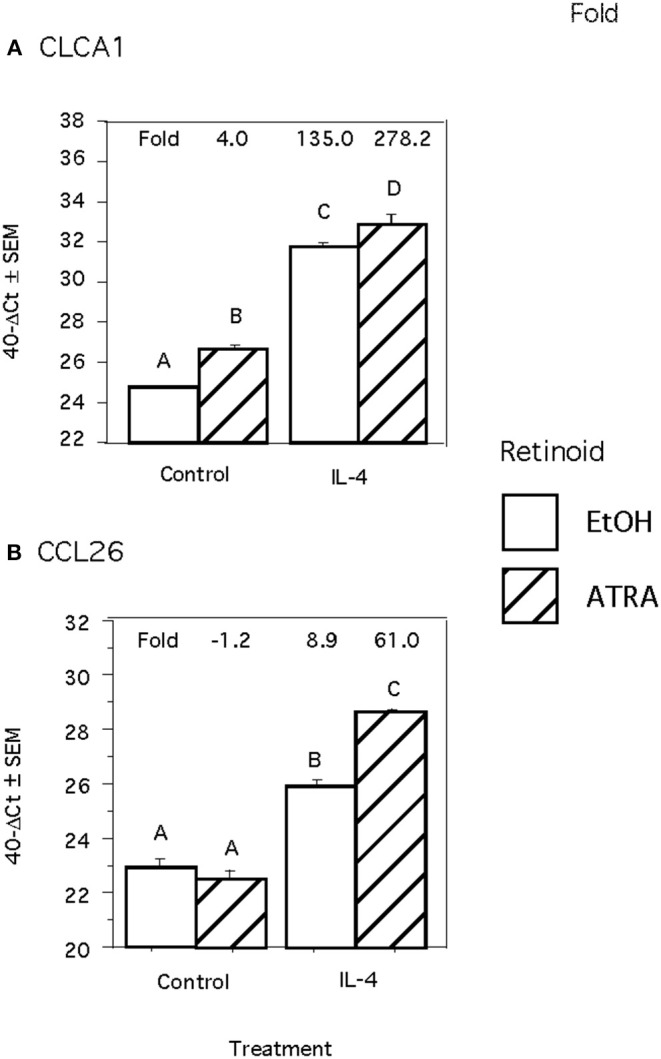
ATRA and IL-4 synergistically induce CLCA1 and CCL26 mRNA Induction in IPEC-J2 Cells. IPEC-J2 cells were treated with ± EtOH or 100 nM ATRA for 18 h then treated with 5.0 ng/ml of porcine IL-4 for 24 h. Messenger RNA of CLCA1 **(A)** and CCL26 **(B)** were analyzed as indicated in Materials and Methods using RPL32 as an internal housekeeping gene. Data were compared by 2-way ANOVA. Treatment with IL-4 alone leads to increased expression of CLCA1 and CCL26 while ATRA further increased expression of both. Means with non-matching superscripts are significantly different at *p* < 0.05 [(*n* = 4 per group), 2 technical replicates].

Because we previously observed increased IL-4 receptor (IL-4R) gene expression in porcine alveolar Mφs treated with ATRA, we sought to determine if porcine epithelial cells responded similarly. Treatment of IPEC1 and IPEC-J2 cells with ATRA upregulated IL-4R mRNA by 5.1 and 6.5-fold, respectively (Figure 1). Since STAT6 mediates the majority of IL-4R signaling, we tested the effect of ATRA pretreatment of IPEC1 and IPEC-J2 cells on IL-4 induced phosphorylation of STAT6. We found that IL-4-induced STAT6 phosphorylation was increased by pretreatment with ATRA in both IPEC1 and IPEC-J2 cells (Figure 2). In addition, 100 nM ATRA pretreatment synergistically increased IL-4 induced CLCA1 and CCL26 expression in IPEC-J2 cells that had been pre-treated with (Figure 3).

We next wanted to determine if the synergistic effect of ATRA and IL-4 treatment on porcine Mφs extended to human Mφs by conducting a series of experiments with the human Mφ cell line, THP-1. Initially we determined whether IL-4R or IRF4 mRNA or proteins were induced by ATRA alone as we observed in porcine Mφs or in the case of IL-4R, porcine epithelial cells. We saw no increase in IL4R mRNA or protein at 1, 2, 4 8, 24, and 48 h. We saw no expression of IRF4 by IL-4 at the mRNA or protein level, despite ample expression of IRF4 in our control IRF4 lysate and GAPDH expression in the samples (data not shown). The expression of CCL2 and TGM2 mRNA and protein, in response to ATRA, were also tested. ATRA induced CCL2 (Table 2) and TGM2 (Table S1) mRNA (at 1, 4, 8, 24, and 48 h,); however, only CCL2 protein (at 8 and 48 h) was significantly induced by ATRA.

**Table 2:** ATRA increases CCL2 mRNA and protein expression.

		**Control**	**ATRA**		
	**Time**	**Adj. mean CT ± SD**	**Adj. mean CT ± SD**	**Fold change**	**Significance**
**RNA**					
	1 h	32.6 ± 0.2	34.1 ± 0.7	2.9	0.0207
	2 h	33.0 ± 0.9	33.3 ± 1.0	1.3	NS
	4 h	33.0 ± 0.2	34.8 ± 0.1	3.6	<0.0001
	8 h	32.0 ± 0.2	34.7 ± 0.4	6.7	0.0006
	24 h	31.9 ± 0.3	33.9 ± 0.0	4.1	0.0032
	48 h	31.4 ± 0.1	32.4 ± 0.4	2.0	0.0171
		**Control**	**ATRA**		
	**Time**	**pg/ml** **±** **SD**	**pg/ml** **±** **SD**	**Fold change**	**Significance**
**PROTEIN**					
	1 h	13 ± 3	8 ± 2	−1.6	NS
	2 h	46 ± 0.2	59 ± 21	1.3	NS
	4 h	56 ± 22	51 ± 16	−1.1	NS
	8 h	141 ± 6	406 ± 32	2.9	0.0002
	24 h	139 ± 20	267 ± 193	1.9	NS
	48 h	247 ± 15	176 ± 6	−1.4	0.0017

*THP-1 cells were treated with ± EtOH or 100 nM ATRA for 1, 2, 4, 8, 24 or 48 h. mRNA was determined by real-time PCR and protein levels determined by ELISA. Expression and protein data were compared by student t-test at each time point [1 technical replicate, (n = 3 per group)]*.

Previous experiments indicated that CCL13 mRNA was the highest mRNA induced by IL-4 in THP-1 cells (39, 40), so we used CCL13 mRNA to determine the ED50 of IL-4 in our system. We estimated that 10 ng/mL was the ED50 (Table S2) and this concentration was used to assess the induction of previously described IL-4 induced genes under our cell culture conditions. Multiple genes, including CCL2, CCL11, CCL17, CCL26, CD209, CD274, fibronectin (FN), Interleukin 13 receptor, alpha 2 (IL13RA2), Mannose receptor; C type 1 (MRC1), neurotrophic tyrosine kinase, receptor, type 1 (NTRK1) and TGM2 were induced to various degrees by IL-4 at 24 and 48 h (data not shown). CCL22 and CCL18 mRNA were inconsistently induced while plasminogen activator, tissue type (PLAT) and Triggering receptor expressed on myeloid cells 2 (TREM2) did not change, and expression of Cytochrome b reductase 1 (CYBB) was reduced by IL-4 (data not shown).

**Figure 4:**
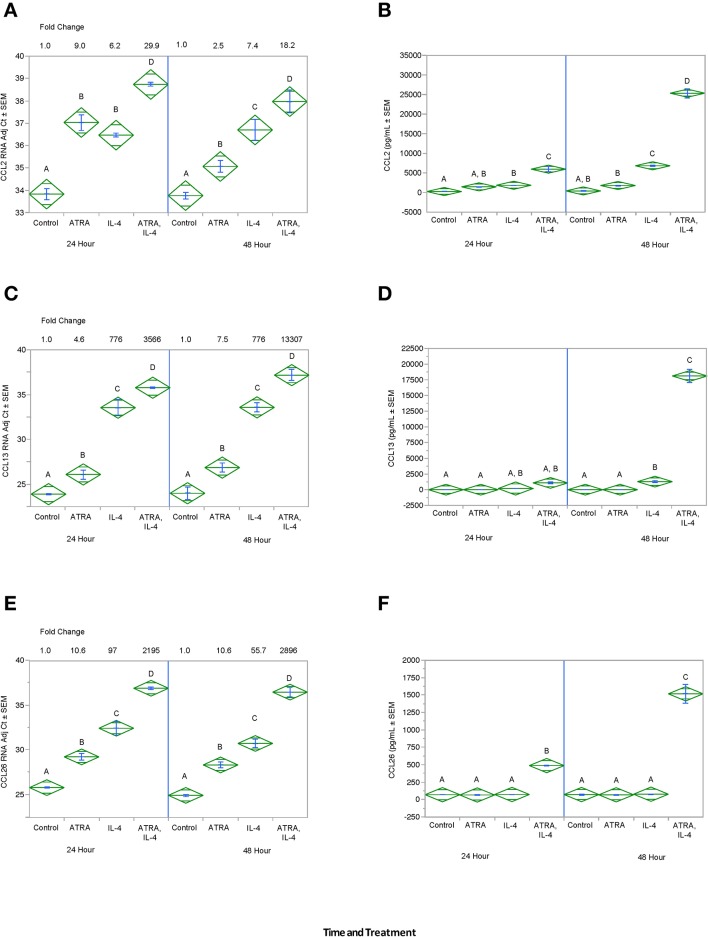
ATRA and IL-4 synergistically induce CCL2, CCL13, and CCL26 mRNA and protein in THP-1 cells. THP-1 cells were treated with ± EtOH or 100 nM ATRA for 18 h and treated with 10.0 ng/ml of human IL-4 for 24 or 48 h. mRNA was determined by real-time PCR and protein levels determined by ELISA for CCL2 **(A,B)**, CCL13 **(C,D)**, and CCL26 **(E,F)**. ANOVA for all the assays had a significance level of *p* < 0.0001. Means with non-matching superscripts are significantly different at *p* < 0.05. [CCL13 and CCL26 (*n* = 3 per group), 3 technical replicates CCL2 (*n* = 3 per group), 2 technical replicates].

**Figure 5:**
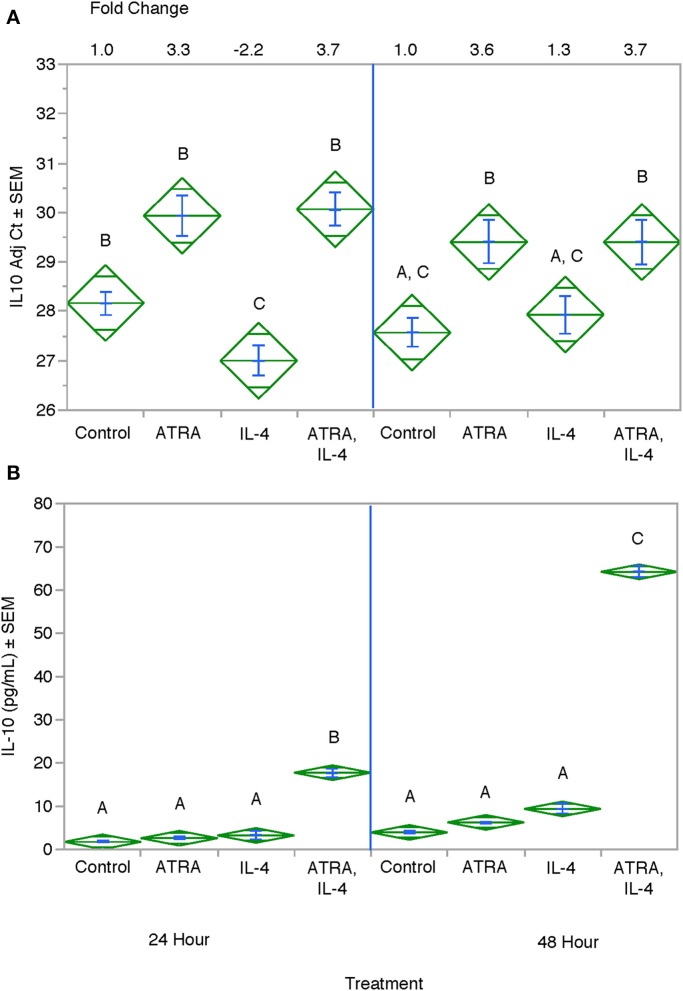
Effect of ATRA and IL-4 on IL-10 mRNA and protein expression in THP-1 cells. THP-1 cells were treated with ± EtOH or 100 nM ATRA for 18 h and treated with 10.0 ng/ml of human IL-4 for 24 or 48 h. mRNA **(A)** was determined by real-time PCR and protein levels **(B)** determined by ELISA. ANOVA for all the assays had a significance level of *p* < 0.0001. Means with non-matching superscripts are significantly different at *p* < 0.05. [(*n* = 3 per group), 2 technical replicates].

**Figure 6:**
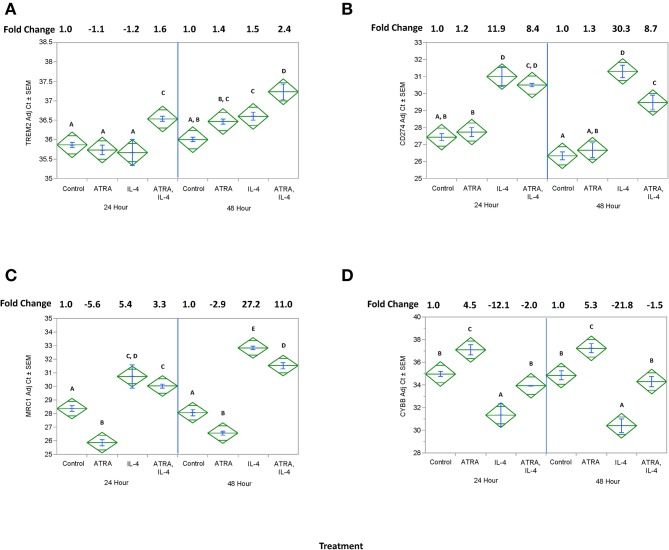
Effect of ATRA and IL-4 on TREM2, CD274, MRC1, and CYBB mRNA expression in THP-1 cells. THP-1 cells were treated with ± EtOH or 100 nM ATRA for 18 h and treated with 10 ng/ml of human IL-4 for 24 or 48 h. mRNA for TREM2 **(A)**, CD274 **(B)**, MRC1, **(C)** or CYBB **(D)** was determined by real-time PCR. ANOVA for all the assays had a significance level of *p* < 0.0001. Means with non-matching superscripts are significantly different at *p* < 0.05. [(*n* = 3 per group), 2 technical replicates].

We then tested the effect of 24 h, 100 nM ATRA pretreatment followed by treatment with 10 ng/ml IL-4 on multiple IL-4-stimulated mRNAs and proteins in THP-1 cells. We found that ATRA and IL-4 had individual and synergistic effects on CCL2, CCL13, and CCL26 mRNA expression (Figures 4A,C,E, respectively), but only the combined action of ATRA and IL-4 induced a significant increase in detectable levels of CCL2, CCL13, and CCL26 proteins (Figures 4B,D,F, respectively). ATRA and IL-4 had a synergistic effect on IL-10 protein levels; however, there was little correlation between mRNA and protein expression (Figures 5A,B, respectively). TREM2 was significantly induced above control levels in ATRA and IL-4 treated cells at 48 h (Figure 6A). CD274 (Figure 6B) and MRC1 (Figure 6C) mRNA were stimulated by IL-4, but inhibited by ATRA, while CYBB mRNA was inhibited by IL-4 and stimulated by ATRA (Figure 6D). There was a trend toward ATRA increasing IL-4 induced CCL11, CCL18, and CCL22 expression at the mRNA and protein level (data not shown), but these effects were not consistent among experiments. Interestingly, the expression of CYP26A1 was increased in the IL-4 and ATRA-treated groups vs. the ATRA-treated groups at both time periods (Figure S1).

**Figure 7:**
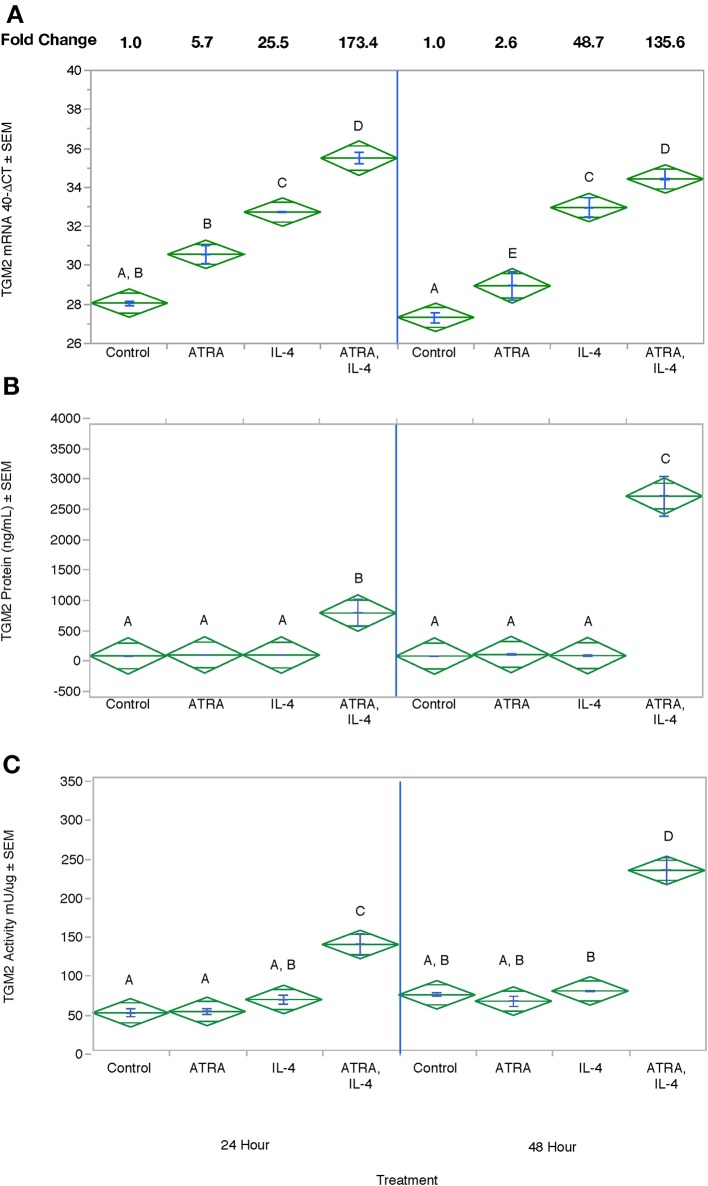
The combination of ATRA and IL-4 increase TGM2 mRNA, protein and enzyme activity in THP-1 Cells. THP-1 cells were treated with ± EtOH or 100 nM ATRA for 18 h and treated with 10 ng/ml of human IL-4 for 24 or 48 h. TGM2 mRNA levels **(A)** were determined by real-time PCR, protein levels **(B)** by ELISA, and enzyme activity by a colorimetric assay **(C)**. ANOVA for all 3 assays had a significance level of *p* < 0.0001. Means with non-matching superscripts are significantly different at *p* < 0.05. [(*n* = 3 per group), 2 technical replicates].

We then tested the effect of 24 h, 100 nM M ATRA pretreatment on IL-4-induced TGM2 mRNA, protein and enzyme activity from THP-1 cells. Similar to the induction of CCL2, CCL13, and CCL26, ATRA and IL-4 had individual and synergistic effects on TGM2 mRNA expression (Figure 7A) but protein levels and enzyme activity were detected only in the groups treated with the combination of IL-4 and ATRA (Figures 7B,C respectively).

**Figure 8:**
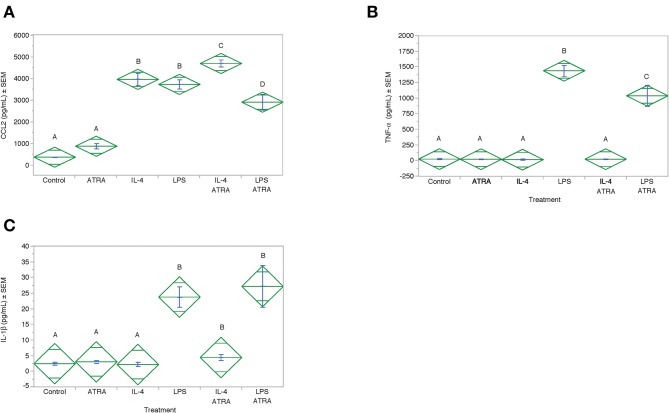
ATRA increased IL-4-induced, but not LPS-induced, CCL2 Protein. THP-1 cells were treated with ± EtOH or 100 nM ATRA for 18 h and treated with 10 ng/ml of human IL-4 or 10 ng/mL *Escherichia coli* LPS for 48 h. Protein levels determined by ELISA. ANOVA for all the assays had a significance level of *p* < 0.0001. Means with non-matching superscripts are significantly different at *p* < 0.05. [(*n* = 4 per group), 1 technical replicate]. Protein levels for CCL2 **(A)**, TNF-α **(B)** and IL-1β **(C)** were determined by ELISA.

To determine if CCL2 was an M1 or M2a associated chemokine under our experimental conditions and whether ATRA synergistically acted with LPS to induce CCL2 in a manner analogous to IL-4. CCL2 protein expression was induced by IL-4 and LPS to a similar degree (Figure 8). However, ATRA increased IL-4 but inhibited LPS induced CCL2 protein expression. As expected LPS induced IL-1β and TNF-α production. ATRA had no effect on LPS-induced IL-1β but inhibited LPS-induced TNF-α (Figure 8).

Finally, we determined whether IL-4 or ATRA had an effect on NF-κβ activation under our culture conditions. Using the NF-κβ reporter assay, we determined that ATRA, IL-4 or the combination of the two had no effect on NF-KB activation (Figure S2). LPS-induced robust NF-κβ activation that was inhibited by ATRA.

## Discussion

Our data demonstrated that ATRA treatment enhances an IL-4-induced Th2 response in both porcine epithelial cell lines and a human Mφ-like cell line. These results indicate that, like porcine Mφs, porcine intestinal epithelial cell responses to IL-4 are increased by exposure to ATRA suggesting that this may be a conserved response to ATRA that spans both cell types and species. ATRA induced IL-4R mRNA and that ATRA-treatment of IPEC-1 and IPEC-J2 porcine epithelial cells led to greater IL-4-induced STAT6 phosphorylation. STAT6 is activated by IL-4 and IL-13 and plays a predominant role in the immune system including clearance of helminthic parasites as well as the pathogenesis of allergic disorders like asthma, food allergies, and atopic dermatitis. Synergistic induction of CLCA1 and CCL26 mRNA in response to IL-4 after pre-treatment of IPEC-J2 cells for 24 h with ATRA was observed. These results recapitulate *in vivo* gene expression changes observed in the lungs of *Ascaris suum*-infected pigs fed ATRA. CLCA1 is an IL-4 and IL-13 inducible (41, 42), STAT6-dependent (29) mediator of chloride ion transport/fluid production (43) and mucin/mucus production (44) and is involved in asthma (41, 45). CCL26 is the dominant (46), STAT6-dependant (47) eosinophil chemoattractant in humans.

We extended our observations made in porcine alveolar Mφs to human-derived THP-1 cells. First, the expression of IL-4 induced genes and proteins for CCL2, CCL13, CCL26, IL10, and TGM2 were increased by ATRA, and ATRA potentiated IL-4-induced TGM2 enzymatic activity. Second, IL-4-induced CD274 and MRC1gene expression was inhibited by ATRA, and CYBB was inhibited by IL-4 but stimulated by ATRA. Finally, IL-4 induced FN1 gene expression was not significantly affected by the addition of ATRA (data not shown).

The expression of chemokines by M2a Mφs between rodents and pigs and humans is dramatically different. CCL2, also known as monocyte chemotactic protein-1 (MCP-1), is reportedly a stable M2a marker in mouse (48) but its expression by human M2a is controversial (40, 49, 50). Other chemokines like CCL11, CCL17, and CCL22 appear to be shared between the species (7); however the expression of CCL13 and CCL18 are exclusive to humans. CCL26 is present in the mouse genome but is not responsive to IL-4 (34), presumably because it lacks a STAT6 response element in its promoter (23).

We previously demonstrated that the protein expression of CCL2 was induced by ATRA in anti-CD3 mAb-stimulated human PBMCs (51). In several previous experiments, ATRA or related compounds, increased the level of CCL2/MCP-1 production and stimulated LPS-induced CCL2 from THP-1 cells and primary human Mφs (13). *In vivo*, a decrease in MCP-1 expression was observed in ATRA-treated mice (52). Notably, a decrease in CCL2/MCP-1protein expression was found in feces of VA-treated children infected with *E. coli*, but not VA-treated children infected with *Ascaris*. In pig alveolar Mφs, CCL2 mRNA and protein were downregulated by IL-4 and upregulated by ATRA. In our current experiment, ATRA induced CCL2 protein and mRNA expression and IL-4 and ATRA synergistically upregulated CCL2 protein in THP-1 cells.

There are other differences between our previous and current findings. In porcine alveolar Mφs, CYB mRNA was downregulated by both ATRA and IL-4. Although it was downregulated in THP-1 cells, it was upregulated by ATRA. CCL18 and CCL22 mRNA and protein were weakly upregulated by IL-4 and inconsistently regulated by ATRA. CCL11 mRNA and protein were strongly upregulated by IL-4 and inconsistently regulated by ATRA. In pig Mφs, CD209, IL13RA2, and NTRK1 were three of the most highly expressed genes that were modestly induced by IL-4 in THP-1 cells (data not shown). Unlike pig Mφs, PLAT mRNA was not affected by IL-4 or ATRA. It is likely that the two very different cell origins of a primary, terminally differentiated, lung-derived pig Mφs vs. an *in vitro* differentiated, THP-1 tumor cell line may account for some of these differences.

Several indirect lines of evidence suggest that the vitamin D receptor (VDR), NF-KB or IL-10 may not play a role in the potentiation of IL-4 signaling by ATRA in Mφs or epithelial cells. Unlike pig Mφs and a previous study using THP-1 cells, the VDR was not induced by ATRA under our cell culture conditions (data not shown). Vitamin D3 (VD3) was also without effect on CCL13 or CCL26 mRNA expression, further suggesting that the obligatory involvement of the VDR in the action of ATRA on M2a polarization is unlikely. Although inhibition of NF-KB is likely involved in the inhibition of M1 polarization by ATRA, the involvement of NF-KB in the M2a polarization is also unlikely as ATRA had no effect on NF-KB activation. IL-10 levels correlated with protein levels for CCL2, CCL13, and CCL26 and it is tempting to speculate that it plays a direct role; however, several lines of evidence contradict this assumption. First, we did not see a change in IL-10 mRNA or protein levels in our pig Mφs or experiments with epithelial cells, yet we still observed potentiation of IL-4 signaling. Although CD274, or programmed cell death 1 ligand 1 (PDCD1LG1), is a known target of IL-10 in Mφs (53), its expression was not correlated with IL-10 levels.

CD274 (PDCD1LG1) was induced by IL-4 in our previous studies in porcine alveolar Mφs where we observed potentiation of expression by ATRA. However, in the current experiments, we observed the opposite since ATRA down regulated IL-4-induced CD274 expression. In a previous experiment, it was reported that VD3 induced the mRNA expression of CD274 in THP-1 cells (54). This occurs only in human cells due to a primate-specific VDRE element in the promoter (54). We observed induction of CD274 by VD3 in THP-1 cells, but its expression was not influenced by ATRA (data not shown).

Another interesting observation was the induction of mRNA for CYP26A1, the enzyme responsible for ATRA catabolism and a sensitive indicator of ATRA bioactivity activity (55, 56), by ATRA in THP-1 cells that were also treated with IL-4. This is in contrast to ATRA-induced CYP26A1 expression that was significantly reduced in THP-1 cells that were also treated with LPS (15). These data suggest that there is reciprocal regulation of CYP26A under M1 and M2a polarizing conditions and that the bioactivity and disposal of ATRA may be facilitated by IL-4.

In conclusion, these data provide important mechanistic information regarding the nutritional role of vitamin A in infection and inflammation suggested from the co-induction of the M2a markers CCL13 and CCL26 along with the M2c marker IL-10. The data also extend the activity of ATRA and IL-4 found in pig alveolar Mφs to pig epithelial cells and human Mφs. The current findings provide potential mechanisms whereby ATRA may contribute to a reduction of inflammation, in humans, and further reinforce our previous finding that ATRA may stimulate a distinct form of alternative activation of Mφs in addition to priming certain aspects of M2a differentiation.

## Data Availability Statement

The datasets generated for this study are available on request to the corresponding author.

## Author Contributions

HD and CC designed the studies and wrote the manuscript. HD and CC performed the major experiments. AS, LC, QP, and JU performed additional experiments. All authors read participated in the revision and approved the final manuscript.

## Conflict of Interest

The authors declare that the research was conducted in the absence of any commercial or financial relationships that could be construed as a potential conflict of interest.

## Abbreviations

AM: Alveolar Macrophage
AAM: Alternatively activated Mφs
ATRA: All-trans-retinoic acid
CCL: Chemokine (C-C motif) ligand
IL1RN: Interleukin 1 receptor antagonist
Mφ: Macrophage
MDM: Monocyte-derived Mφ
PBS: Phosphate Buffered Saline
VDR: Vitamin D3 receptor
VD3: Vitamin D (1,25- dihydroxyvitamin D3).
