# Supplementary material for: Potentiation of IL-4 Signaling by Retinoic Acid in Intestinal Epithelial Cells and Macrophages—Mechanisms and Targets
Source: Front Immunol. 2020 May 5;11:605. doi: 10.3389/fimmu.2020.00605 (PMC7214669; doi:10.3389/fimmu.2020.00605)
Supplement: Supplementary file 1 [file Image_1.pdf]

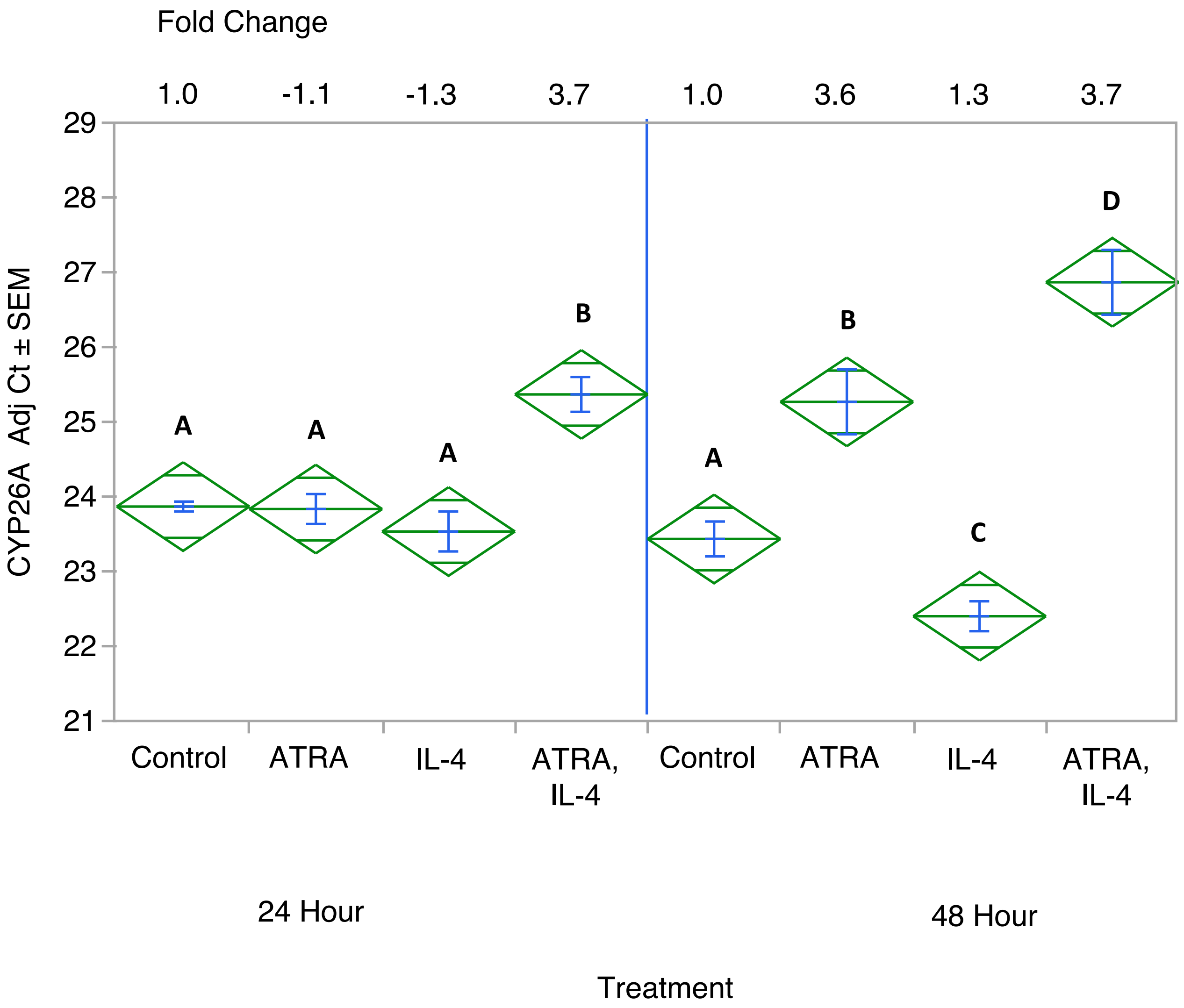


**Figure 1S. Effect of ATRA and IL-4 on CYP26A1 mRNA expression in THP-1 cells.**

THP-1 cells were treated with +/- EtOH or 10-7 M ATRA for 18 h and treated with 10 ng/ml of human IL-4 for 24 or 48 h. mRNA was determined by real-time PCR. ANOVA had a significance level of $p < 0.0001$. Means with non-matching superscripts are significantly different at $p < 0.05$. [(n = 3 per group), 2 technical replicates].
