# Supplementary material for: Potentiation of IL-4 Signaling by Retinoic Acid in Intestinal Epithelial Cells and Macrophages—Mechanisms and Targets
Source: Front Immunol. 2020 May 5;11:605. doi: 10.3389/fimmu.2020.00605 (PMC7214669; doi:10.3389/fimmu.2020.00605)
Supplement: Supplementary file 2 [file Image_2.pdf]

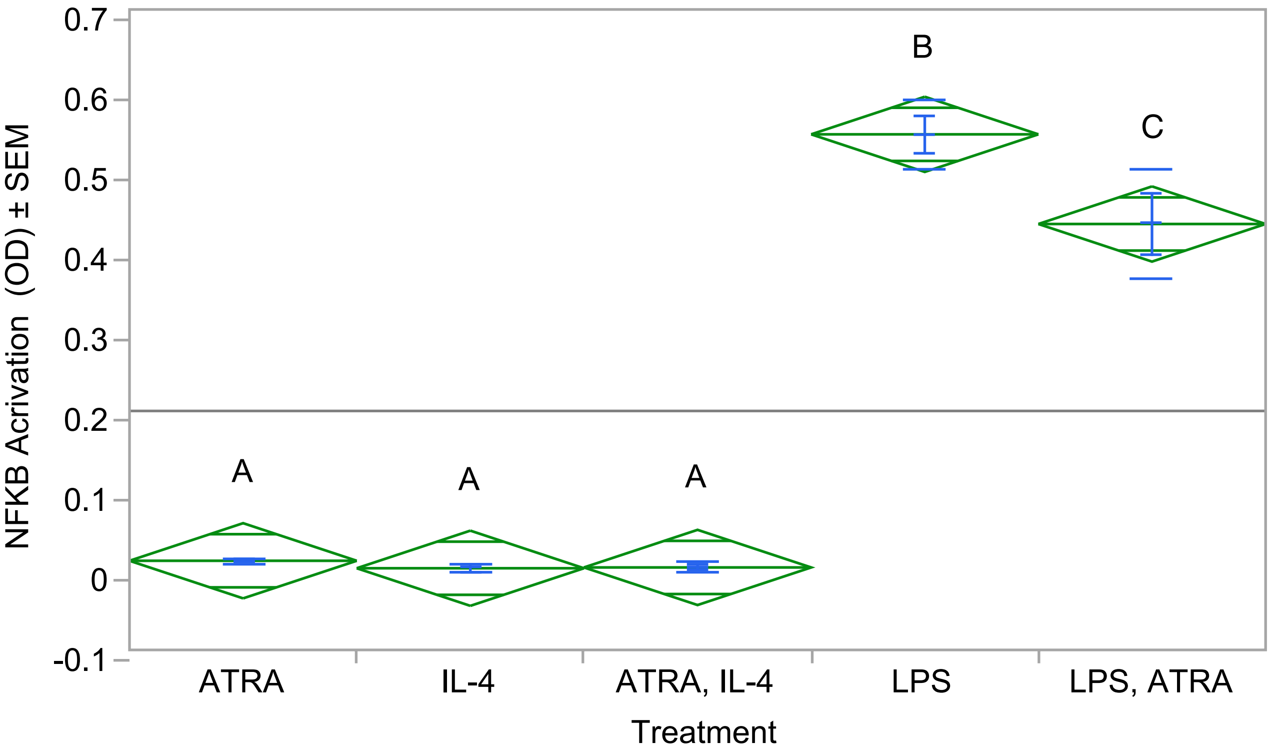

Figure 2S. Effect of ATRA, IL-4 and LPS on NF-κβ Activation of THP-1 cells.

Differentiated THP1-XBlue™ cells were pretreated with EtOH or ATRA, as described above. Cells were then treated with IL4 (10 ng/mL) or LPS (10 ng/mL) for 24h. SEAP production in cell culture supernatants was measured to assess NF-κB activity as described in the Materials and Methods section. ANOVA for all the assays had a significance level of $p < 0.0001$. Means with non-matching superscripts are significantly different at $p < 0.05$. [(n = 3 per group), 1 technical replicate].
